# Cavin-2 loss exacerbates hypoxia-induced pulmonary hypertension with excessive eNOS phosphorylation and protein nitration

**DOI:** 10.1016/j.heliyon.2023.e17193

**Published:** 2023-06-11

**Authors:** Takeru Kasahara, Takehiro Ogata, Naohiko Nakanishi, Shinya Tomita, Yusuke Higuchi, Naoki Maruyama, Tetsuro Hamaoka, Satoaki Matoba

**Affiliations:** a Department of Cardiovascular Medicine, Graduate School of Medical Science, Kyoto Prefectural University of Medicine, Kyoto 602-8566, Japan; b Department of Pathology and Cell Regulation, Graduate School of Medical Science, Kyoto Prefectural University of Medicine, Kyoto 602-8566, Japan

**Keywords:** Pulmonary hypertension, Endothelial cells, Caveolin-1, eNOS, Nitration, PKG, Cavin-2

## Abstract

Pulmonary hypertension (PH) is associated with a poor prognosis even in recent years. Caveolin-1 (CAV1), a caveolae-associated protein, is a causal gene in PH. Cavin-2, one of the other caveolae-associated proteins, forms protein complexes with CAV1 and influences each other's functions. However, the role of Cavin-2 in PH has not been thoroughly investigated. To clarify the role of Cavin-2 in PH, we exposed Cavin-2-deficient (Cavin-2 KO) mice to hypoxia. A part of the analyses was confirmed in human pulmonary endothelial cells (HPAECs). After 4-week 10% O_2_ hypoxic exposure, we performed physiological, histological, and immunoblotting analyses. Right ventricular (RV) systolic pressure elevation and RV hypertrophy were exacerbated in Cavin-2 KO mice with hypoxia-induced PH (Cavin-2 KO PH mice). The vascular wall thickness of pulmonary arterioles was aggravated in Cavin-2 KO PH mice. Cavin-2 loss reduced CAV1 and induced sustained endothelial nitric oxide synthase (eNOS) hyperphosphorylation in the Cavin-2 KO PH lungs and HPAECs. NOx production associated with eNOS phosphorylation was also increased in the Cavin-2 KO PH lung and HPAECs. Furthermore, the nitration of proteins, including protein kinase G (PKG), was raised in the Cavin-2 KO PH lungs. In conclusion, we revealed that Cavin-2 loss exacerbated hypoxia-induced PH. Our results suggest that Cavin-2 loss leads to sustained eNOS hyperphosphorylation in pulmonary artery endothelial cells via CAV1 reduction, resulting in Nox overproduction-mediated nitration of proteins, including PKG, in smooth muscle cells.

## 1 Introduction

Pulmonary hypertension (PH) is a progressive disease that results in right heart dysfunction and is associated with a poor prognosis even in the recent past [1]. Some PH has a genetic involvement, and various causal genes have been identified so far [2]. Many causal genes are related to bone morphogenetic protein receptor type 2 (BMPR2), a member of the transforming growth factor-β superfamily. However, some known heritable PH causative genes are involved in PH through mechanisms independent of BMPR2-associated signals; caveolin-1 (CAV1), a caveolae-associated protein, has a BMPR2-independent mechanism in PH development [3,4].

Caveolae are 50–100 nm flask-shaped microdomains on the plasma membrane involved in several critical cellular processes, including signal transduction, endocytosis, and cholesterol homeostasis [[5], [6], [7]]. Caveolins and cavins are important for caveolae formation, and CAV1 is essential for caveolae formation in endothelial cells [8]. CAV1 also inhibits endothelial nitric oxide synthase (eNOS) activity by binding to the eNOS on the caveolae of the endothelial cells [9]. Loss of CAV1 causes excessive eNOS phosphorylation and induces nitration of intracellular proteins, including protein kinase G (PKG) in smooth muscle cells, involved in vasoconstriction and vascular remodeling [9]. These nitrations contribute to the development of PH.

Cavins, caveolae-coating proteins, also cooperate with the caveolin to regulate the caveolae's shape and function [[10], [11], [12], [13], [14], [15]]. Cavin-2, one of the cavins, is highly expressed in normal adipose, lung, and heart tissues in mice and humans [8,16,17], is abundantly expressed in endothelial cells in these tissues. Cavin-2 is co-localized with CAV1 and Cavin-1 in those tissues: Cavin-2 forms a complex with CAV1 to regulate the formation and morphology of caveolae [18]. Previously, Cavin-2 protein expression was reported to decrease in blood outgrowth endothelial cells isolated from patients with PAH [19]. Loss of Cavin-2 decreases CAV1 and the number and depth of caveolase in the endothelial cells of the lung [8,19]. Furthermore, an association of eNOS with Cavin-2 in endothelial cells was shown upon hypoxic stimulation [20]. However, the role of Cvain-2 in PH has not been fully extracted and needs to be confirmed *in vivo* models.

We here highlight the importance of Cavin-2 loss in the mechanism of PH pathogenesis. Our results suggest that Cavin-2 loss leads to excessive eNOS hyperphosphorylation in pulmonary artery endothelial cells (PAECs) via CAV1 reduction, resulting in NOx overproduction-mediated nitration of proteins, including PKG in smooth muscle cells.

## 2 Material and methods

### 2.1 Animals

The generation of Cavin-2 knockout (KO) mice was as we previously described [11]. The wild-type (WT) and Cavin-2 KO mice were on a C57BL/6 J genetic background. All aspects of animal care and experimentation performed in this study were approved by the Institutional Animal Care and Use Committee of Kyoto Prefectural University of Medicine (30–157).

### 2.2 Cell culture and hypoxia experiments in vitro

Human pulmonary arterial endothelial cells (HPAECs) were purchased from Lonza and cultured in EGM-2 medium (Lonza) supplemented with EGM-2 SingleQuots (Lonza) according to the manufacturer's instructions. HPAECs were grown to 80–90% confluence and subsequently incubated under normoxic (21% O_2_–5% CO_2_) or hypoxic (1% O_2_–5% CO_2_-balance N_2_) conditions at 37 °C for 48 h in a hypoxic chamber (Astec, Fukuoka, Japan).

### 2.3 Gene silencing through RNA interference

**Fig. S1:**
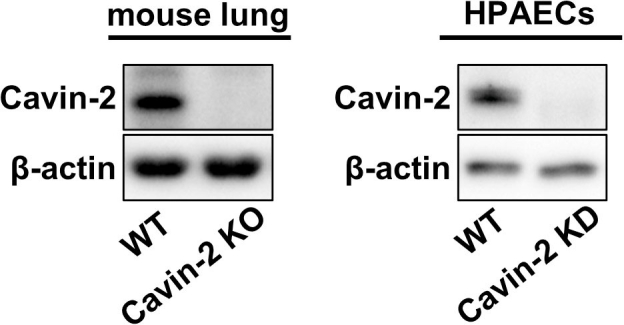
Representative Western blot images of Cavin-2 of mouse lung tissue (left) and HPAECs (right).

Human Cavin-2-specific and control small interfering RNA (siRNA) duplex oligonucleotides (Stealth RNA interference (RNAi)) were purchased from Invitrogen. SiRNAs were transiently transfected into HPAECs using Lipofectamine RNAi MAX reagent (Invitrogen) according to the manufacturer's protocol. The medium was changed 4 h post-transfection and cells were used for assay 72 h after transfection. The siRNA sequences were as follows: *Cavin-2* siRNA (sense, 5′-GGAACAGGCACAGAAGGUACGCUAU-3’; antisense, 5′-AUAGCGUACCUUCUGUGCCUGUUCC-5′). We confirmed sufficient Cavin-2 knockdown in HPAECs 72 h after siRNA transfection (Fig. S1).

### 2.4 Hypoxic mouse model of PH

WT mice and Cavin-2 KO mice were exposed to normobaric hypoxia (10% O_2_) in a chamber in which oxygen was tightly regulated by the oxygen controller ProOx110 (KYODO International, Kanagawa, Japan) for 4 weeks [21]. Age- and sex-matched littermates were exposed to identical conditions in normoxia and served as controls.

### 2.5 Physiological assessment of PH

A thoracotomy was performed under positive pressure ventilation and general anesthesia with 1% isoflurane. Right ventricular (RV) systolic pressure (RVSP) was measured by inserting a catheter directly into the RV. We only adopted waveforms that were maintained for 1 min. The lungs and heart were subsequently removed. The RV weight ratio to left ventricular (LV) including septal (S) weight (RV/LV + S, Fulton's index) and the lung weight ratio to tibial length (LW/TL) were measured to evaluate RV hypertrophy.

### 2.6 Transmission electron microscopy and quantitation

Fourteen-weeks-old mouse lungs were fixed with 2% glutaraldehyde in 0.1 M cacodylate buffer, post-fixed with 2% OsO_4_, and stained with uranyl acetate and lead citrate. Microtome sections were examined under an H-7100 transmission electron microscope (HITACHI, Tokyo, Japan) and photographed at a magnification of 30,000. Caveolae were identified by their characteristic flask shape and location at or near the plasma membrane [14].

### 2.7 Histological analyses

Pulmonary vascular remodeling was assessed by measuring the medial thickness of alveolar/distal pulmonary vessels of 40–100 mm in diameter from lung sections immunostained with α smooth muscle actin (αSMA) (Sigma) and CD31 (R&D systems) antibodies. Percent wall thickness is expressed as the medial wall area (the area between the internal and external lamina) divided by the area of the vessel (the area between the external lamina).

### 2.8 Lung tissue protein sampling

Whole lung tissue was homogenized in lysis buffer [50 mM Tris-HCl (pH 7.5), 150 mM NaCl, EDTA (pH 9.0), octylphenol ethoxylate, protease and phosphatase inhibitor cocktails]. The lysate was then centrifuged at 15,000 rpm for 10 min at 4 °C. Protein samples in the supernatant were used for Western blotting.

### 2.9 Western blotting

Protein samples from whole lung tissues or cultured cells were subjected to Western blot analysis with antibodies against Cavin-2 (Proteintech), Caveolin-1 (Santa Cruz Biotechnology), phosphorylated eNOS (Cell Signaling), eNOS (Cell Signaling), nitrotyrosine (Abcam), PKG-1α (Enzo), phospho-Smad1 (Ser463/465)/Smad5 (Ser463/465)/Smad9 (Ser465/467) (Cell Signaling), Smad1 (Cell Signaling), phospho-MLC2 at Ser19 (Cell Signaling), MLC (Cell signaling), and Gapdh (Abcam). Signal intensities were determined using Image J software (National Institutes of Health, Bethesda, MD). Uncropped data of Western blots are provided in Figs. S7–S9.

### 2.10 RNA extraction and quantitative reverse transcriptase (qRT)-PCR

Total RNA was extracted from hearts using Trizol reagent (Invitrogen) and then treated with DNase I (Qiagen) to remove any residual DNA. Five hundred nanograms of total RNA was converted to cDNA using the High Capacity cDNA Reverse Transcription Kit (Applied Biosystems). Synthesized cDNA was analyzed by kinetic real-time PCR using TaKaRa PCR Thermal Cycler Dice (TAKARA BIO INC., Japan) with Platinum SYBR Green qPCR Super Mix (Invitrogen). Primers used for qRT-PCR are listed in Table S1. *β-actin* or *GAPDH* was used for normalization.

### 2.11 Cycloheximide (CHX) chase assay

HPAECs were cultured in EGM-2 medium with the supplement in the presence of CHX (10 μg/ml; Sigma-Aldrich), which was used to block protein synthesis. Cell lysates were collected at different time points (t = 0, 24, and 48 h) after administration of CHX under normoxic or hypoxic conditions, followed by Western blot analysis for Cavin-2. GAPDH was used for normalization.

### 2.12 Nitrate and nitrite production

For estimating the Nitric oxide (NO) production level in mouse lungs, the production of nitrate and nitrite in homogenized whole lung tissue was measured by Nitrate/Nitrite Colorimetric Assay Kit (Cayman) according to the manufacturer's protocol.

### 2.13 PKG nitration assay

The PKG nitration detection assay was performed by modifying the immunoprecipitation protocol of Aggarwal et al. [22]. Immunoprecipitation was carried out by incubating the same amount of tissue lysates with magnetic beads (Magnosphere MS300/Carboxyl, COSMO BIO, Tokyo, Japan) coated with PKG-1α antibody at 4 °C overnight. Beads were washed with wash buffer (50 mM Tris, pH 8.0, 50 mM NaCl, 1.0% Nonidet P-40, 1 mM NaF) five times. The precipitated proteins were separated by SDS–PAGE, transferred to a polyvinylidene difluoride membrane, and probed with nitrotyrosine antibody. IP efficiency was normalized by re-probing with PKG-1α.

### 2.14 Statistical analysis

All data are presented as mean ± standard error of the mean (SEM). All analyses were performed using GraphPad Prism Software, version 5 (GraphPad, San Diego, CA). Statistical significance was determined by one-way ANOVA for multiple groups, followed by Bonferroni post-hoc analysis. In comparing only two groups, we used the unpaired Student's t-test. A *p*-value <0.05 was considered statistically significant.

## 3 Results

### 3.1 Cavin-2 loss exacerbated hypoxia-induced PH in mice

**Fig. 1:**
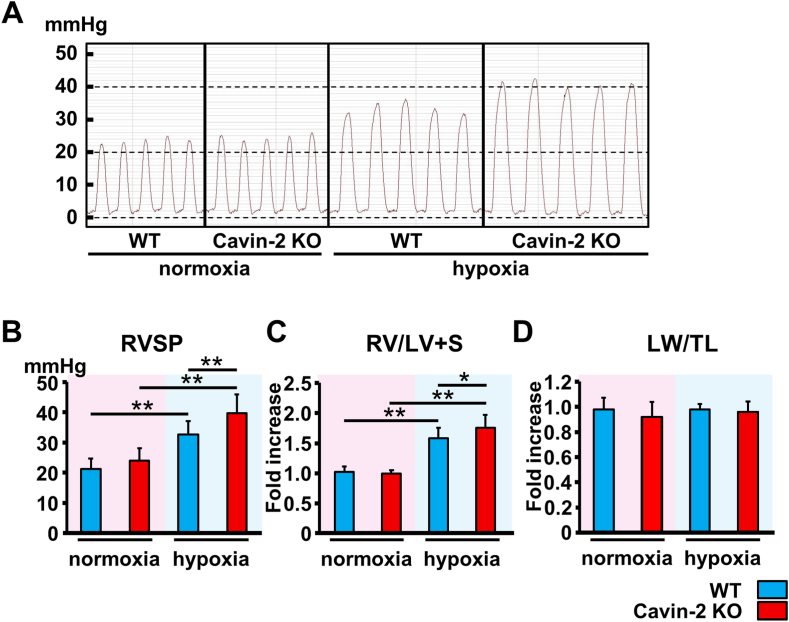
**Cavin-2 loss exacerbates hypoxia-induced pulmonary hypertension.** Representative graphs of the pressures of right ventricle after normoxia (21% O_2_ for 4 weeks) or hypoxia (10% O_2_ for 4 weeks) exposure (**A**) and right ventricular systolic pressure (RVSP) (**B**). (**C**) Relative RV weight was determined as the ratio of the RV weight to the left ventricular (LV) including interventricular septum (S) weight. (**D**) Relative lung weight (LW) was determined as the ratio of the LW to the tibial length (TL). WT normoxia, *n* = 20; Cavin-2 KO normoxia, *n* = 11; WT hypoxia, *n* = 12; Cavin-2 KO hypoxia, *n* = 9. *P < 0.05, **P < 0.01. Data are presented as mean ± SEM. WT, wild-type; KO, knock-out.

To investigate the functional significance of Cavin-2 in the development of PH, we employed a model of mouse PH due to chronic hypoxia [23]. After exposure to normoxic or 10% O_2_ hypoxic condition for 4 weeks, we compared RVSP, RV weight ratio to LV weight including septum (RV/LV + S), and the lung weight ratio to tibial length (LW/TL). WT and Cavin-2 KO mice displayed significant elevations in RVSP with RV hypertrophy after hypoxic exposure but not the normoxic condition (Fig. 1A, B, and 1C). Furthermore, the RVSP elevation and RV hypertrophy after hypoxia were significantly higher and more severe in Cavin-2 KO mice compared to WT mice. There was no significant difference in LW/TL between each group (Fig. 1D).

### 3.2 Cavin-2 loss decreased the number of caveolae in endothelial cells and exacerbated pulmonary vascular remodeling in the PH lung

**Fig. 2:**
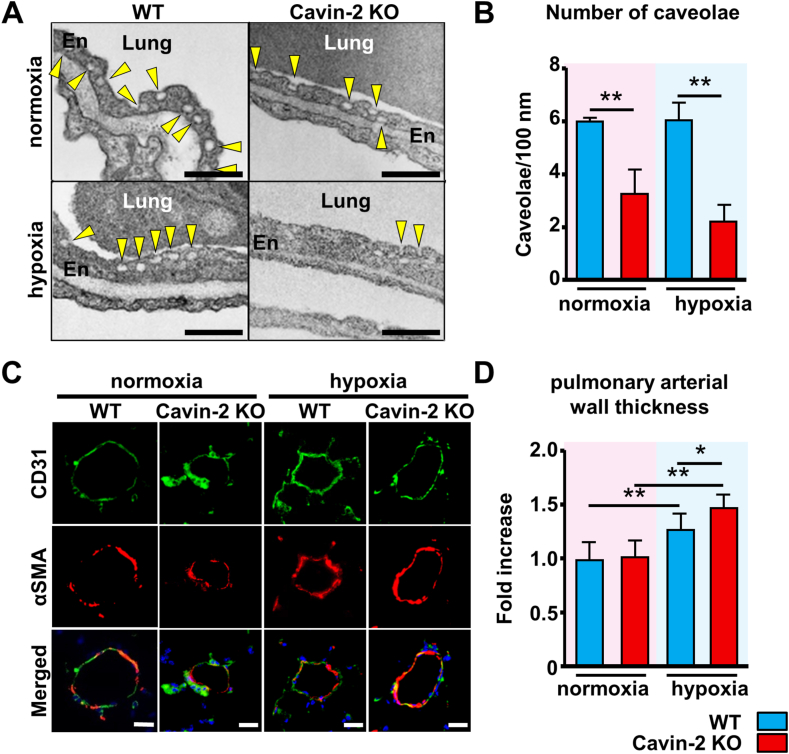
**Cavin-2 loss decreases the number of caveolae in endothelial cells and exacerbates pulmonary vascular remodeling in the chronic hypoxic lung.** (**A**) Representative photos of pulmonary arterial endothelial cells (MPAECs) in the mouse lung tissue by electron microscopy. The scale bar is 320 nm. (**B**) The graph shows the number of caveolae in MPAECs per 100 nm. WT normoxia, *n* = 3; Cavin-2 KO normoxia, *n* = 3; WT hypoxia, *n* = 3; Cavin-2 KO hypoxia, *n* = 3. (**C**) Representative fluorescent images of pulmonary arterial vessels. The scale bar is 20 μm. (**D**) The graph shows the percent wall thickness, which is expressed as the medial wall area divided by the area of the vessel. WT normoxia, *n* = 6; Cavin-2 KO normoxia, *n* = 6; WT hypoxia, *n* = 6; Cavin-2 KO hypoxia, *n* = 6. Twenty small muscularized vessels were randomly selected per mouse lung. *P < 0.05, **P < 0.01. Data are presented as mean ± SEM. En, endothelial cells.

**Fig. S2:**
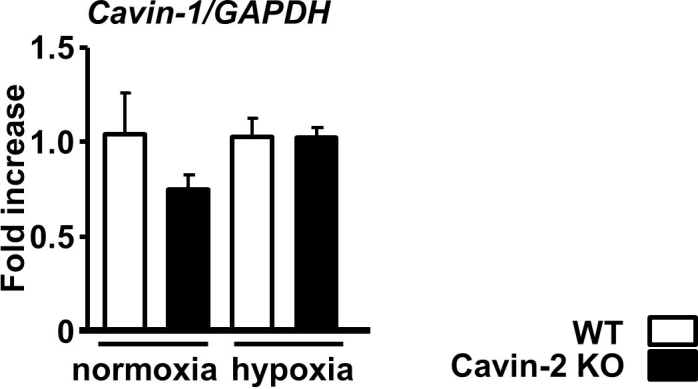
**mRNA expression of Cavin-1 in the lung.** The relative level of expression of Cavin-1 mRNA in WT mouse lung. WT normoxia, n = 3; WT hypoxia, n = 5; KO normoxia, n = 5; KO hypoxia, n = 5. Data are presented as mean ± SEM.

Cavin-2 is a caveolae-related protein and affects caveolae morphology. Therefore, we evaluated the morphology and number of caveolae in mouse pulmonary artery endothelial cells (MPAECs) after chronic hypoxic exposure using by electron microscope. We could not assess the depth of membrane invaginations due to the insufficient amount available for analysis. Still, it looked like the depth of membrane invaginations in MPAECs was slightly shortened under hypoxia (Fig. 2A), and the number of caveolae was significantly decreased in the Cavin-2 KO mice compared to the WT mice (Fig. 2B). The mRNA expression of Cavin-1 was not changed between the WT and Cavin-2 KO mice with and without hypoxia (Fig. S2). A previous report showed that the depth of membrane invaginations in the Cavin-2 KO mice was shortened even under normoxic conditions without a change of Cavin-1 expression [8]. Although their results are not entirely consistent with ours, our results suggest that loss of Cavin-2 affects caveolae morphology, becoming more clarified under hypoxia.

We also evaluated vascular remodeling of the pulmonary arterioles by vascular wall thickness. In PH, the vascular wall thickening of muscularized arterioles is observed [13]. The antibodies of CD31 and αSMA were used to identify the muscularized arteriolar vessels (CD31 (+), αSMA (+)). Under the normoxic conditions, there was no difference in the αSMA-positive area within the pulmonary arterioles between WT and Cavin-2 KO mice (Fig. 2C and D). However, after chronic hypoxic exposure, vascular wall thickening was observed in both WT and Cavin-2 KO mice, and the degree of the thickening in Cavin-2 KO mice was significantly more aggravated than in WT mice (Fig. 2C–D). To confirm the effect of Cavin-2 loss on the vascular endothelium, we also attempted to identify non-muscular arteriolar vessels accurately. Still, we could not achieve this because of the low resolution of the acquired images.

### 3.3 Cavin-2 loss decreased CAV1 and induced excessive eNOS phosphorylation in the PH lung and HPAECs

**Fig. 3:**
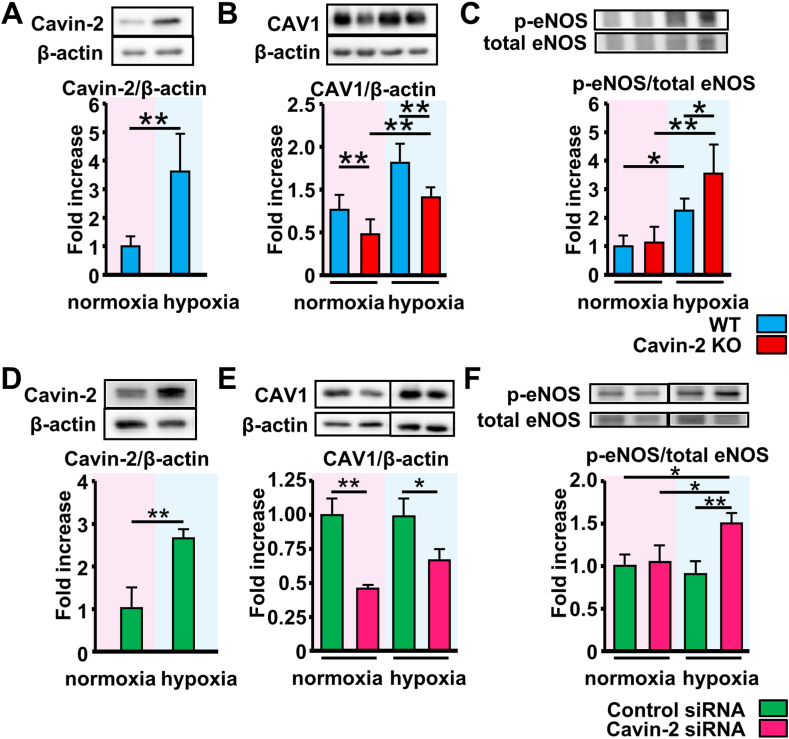
**Cavin-2 loss decreases caveolin-1 expression and induces excessive eNOS phosphorylation in the chronic hypoxic lung and human pulmonary endothelial cells.** The relative levels of expression of Cavin-2 (**A, D**) and caveolin-1 (**B, E**), and eNOS phosphorylation (**C, F**) in mouse lung tissues (**A-C**, *n* = 6 in each group) and human pulmonary artery endothelial cells (HPAECs) (**D-F**, *n* = 33 in each group). The lung samples were collected after exposure to normoxia or hypoxia for 4 weeks. The HPAECs were exposed to normoxia (21% O_2_, 5% CO_2_) or hypoxia (1% O_2_, 5% CO_2_) for 48 h after transfection of siRNAs. The in vitro experiments were performed at least 3 times. *P < 0.05, **P < 0.01. Data are presented as mean ± SEM. Original blots are depicted in the supplement files “Fig. S7” and “Fig. S8”. CAV1, caveolin-1; *p*-eNOS, phosphorylated eNOS.

**Fig. S3:**
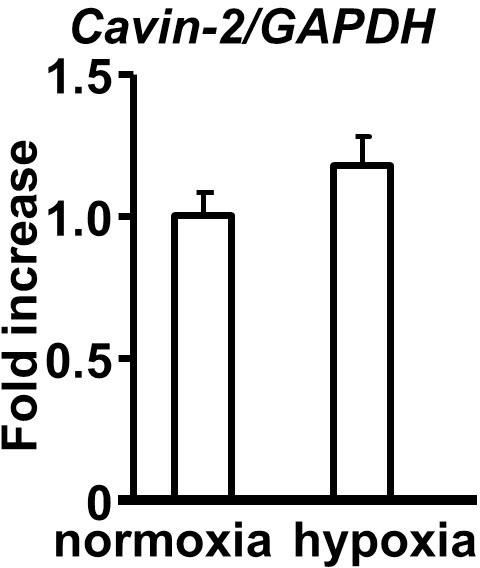
**mRNA expression of Cavin-2 in the lung after hypoxia.** The relative levels of expression of Cavin-2 mRNA in mouse lung tissues (WT normoxia, n = 3; WT hypoxia, n = 5). Data are presented as mean ± SEM.

**Fig. S4:**
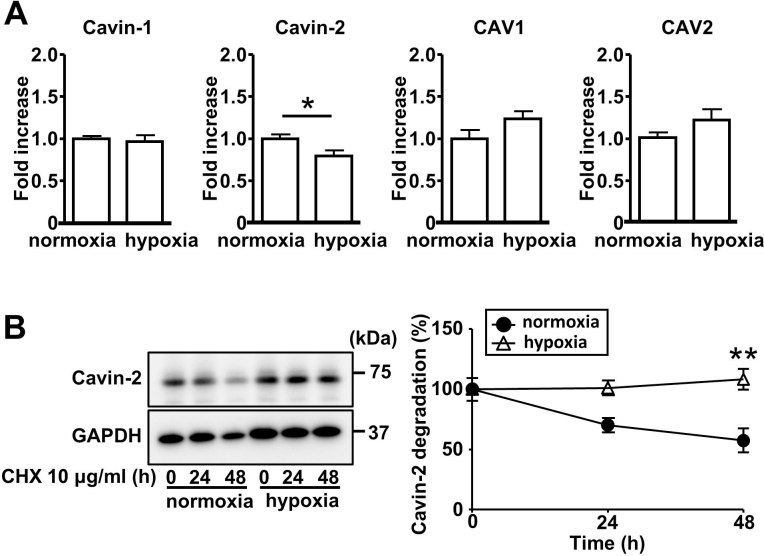
**The mRNA expression levels of caveolar-related genes and protein degradation of Cavin-2 in HPAECs after hypoxic exposure.** (**A**) The relative mRNA expression levels of Cavin-1, Cavin-2, CAV1, and CAV2 in HPAECs (n = 5 in each group). (**B**) The representative Western blot images and quantitative data of Cavin-2 protein degradation by cycloheximide (CHX) chase assay. **P < 0.01, *P < 0.05. Data are presented as mean ± SEM.

We evaluated the protein expression level of Cavin-2 and CAV1 in the lung tissues and the human pulmonary artery endothelial cells (HPAECs). Cavin-2 protein expression was increased in the lung and HPAECs exposed to hypoxia compared to normoxic conditions (Fig. 3A and D). Cavin-2 mRNA expression in the lung and HPAECs was not increased after hypoxic exposure (Figs. S3 and S4A), whereas the degradation of Cavin-2 protein in HPAECs was markedly inhibited under hypoxic conditions (Fig. S4B). CAV1 protein expressions were decreased in the Cavin-2 KO mouse lung and Cavin-2 knockdown (KD) HPAECs and these decreases were maintained after hypoxic exposure (Fig. 3B and E). mRNA expression levels of CAV1, caveolin-2 (CAV2), and Cavin-1 were no affected by hypoxic exposure (Fig. S4A). Reduction of CAV1 increases eNOS phosphorylation. After hypoxic exposure, eNOS phosphorylation was significantly increased in the Cavin-2 KO mouse lung and Cavin-2 KD HPAECs compared to the WT controls (Fig. 3C and F).

**Fig. S5:**
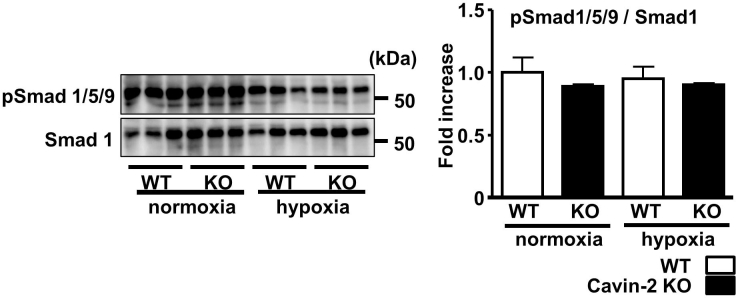
**Smad1/5/9 phosphorylation in the mouse lung after hypoxia.** The relative phosphorylation level of Smad1/5/9 in the mouse lung (n = 3 in each group). Data are presented as mean ± SEM.

We evaluated the effect of Cavin-2 loss on the phosphorylation of Smad2 and Smad1/5/9 in mouse lung tissue because CAV1 has a BMPR2-independent mechanism in PH development [3,4]. Smad2 phosphorylation in the lung tissue was hardly confirmed (data not shown). Smad1/5/9 phosphorylation in the lung tissue was no different among all mice groups (Fig. S5). These results suggest that TGFβ/Samd2 or BMPR2/Smad1/5/9 signaling, like CAV1, may not be central to the mechanisms in Cavin-2 loss-associated PH.

### 3.4 Cavin-2 loss facilitated NOx production and nitration of proteins in the PH lung

**Fig. 4:**
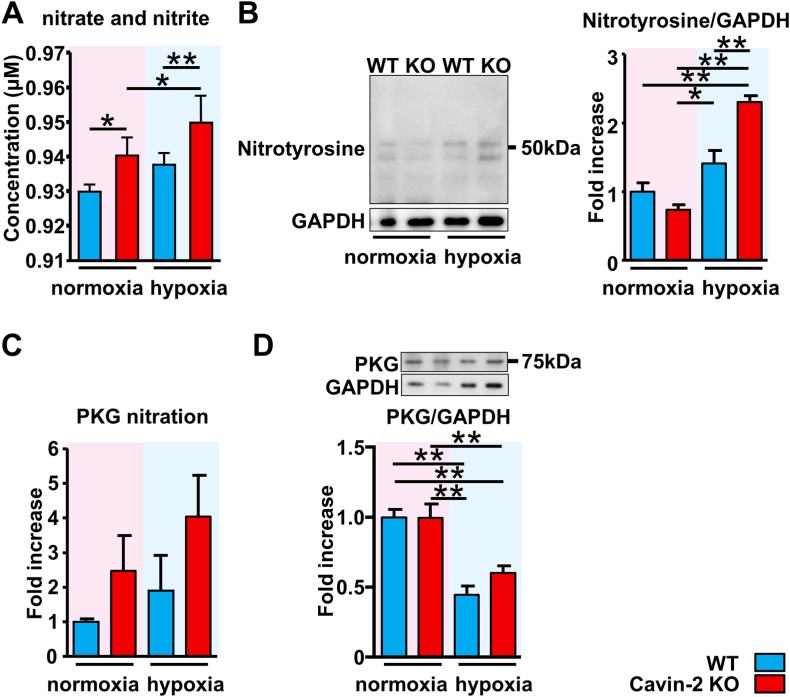
**Cavin-2 loss facilitates NOx production and nitration of proteins in the chronic hypoxic lungs.** (**A**) Nitrate and Nitrite (NOx) production level in the mouse lung tissues. WT normoxia, *n* = 5; Cavin-2 KO normoxia, *n* = 6; WT hypoxia, *n* = 6; Cavin-2 KO hypoxia, *n* = 6. (**B**) Representative Western blot image and quantitative data of nitrotyrosine level in the mouse lung tissues (*n* = 3 in each group). Quantitative data of PKG nitration (**C**) and PKG expression (**D**) in the mouse lung tissues (*n* = 3 in each group). *P < 0.05, **P < 0.01. Data are presented as mean ± SEM. Original blots are depicted in the supplement file “Fig. S9”.

Nitrate and nitrite (NOx) production and nitration in the lung tissues were evaluated because excessive eNOS phosphorylation produces excessive NO in the lung. NOx production increased in the lung of Cavin-2 KO mice compared to those of the WT mice. Hypoxic exposure facilitated the excessive NOx production in the lung of Cavin-2 KO mice (Fig. 4A). Nitrotyrosine is widely used as a nitrosative stress marker because it is produced by modification of protein tyrosine residues by peroxynitrite generated from the reaction of NO and superoxide [24]. Lung nitrotyrosine level was significantly elevated in the Cavin-2 KO mice after hypoxic exposure (Fig. 4B). Since nitration-induced PKG impairment is associated with PH, we also evaluated PKG nitration in the lung. Although not significantly different, the PKG nitration in lung tissue tended to be enhanced in Cavin-2 KO mice, especially after exposure to hypoxia (Fig. 4C). The PKG expression in the lung was significantly decreased after hypoxic exposure. Still, it was unaffected by the Cavin-2 expression level (Fig. 4D).

**Fig. S6:**
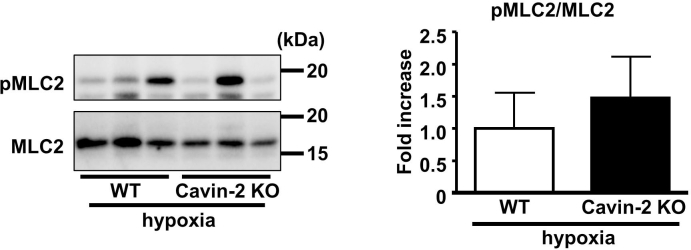
**Phosphorylation of MLC2 in the mouse lung after hypoxia.** The relative phosphorylation level of MLC2 in the mouse lung (n = 3 in each group). Data are presented as mean ± SEM.

**Fig. S7:**
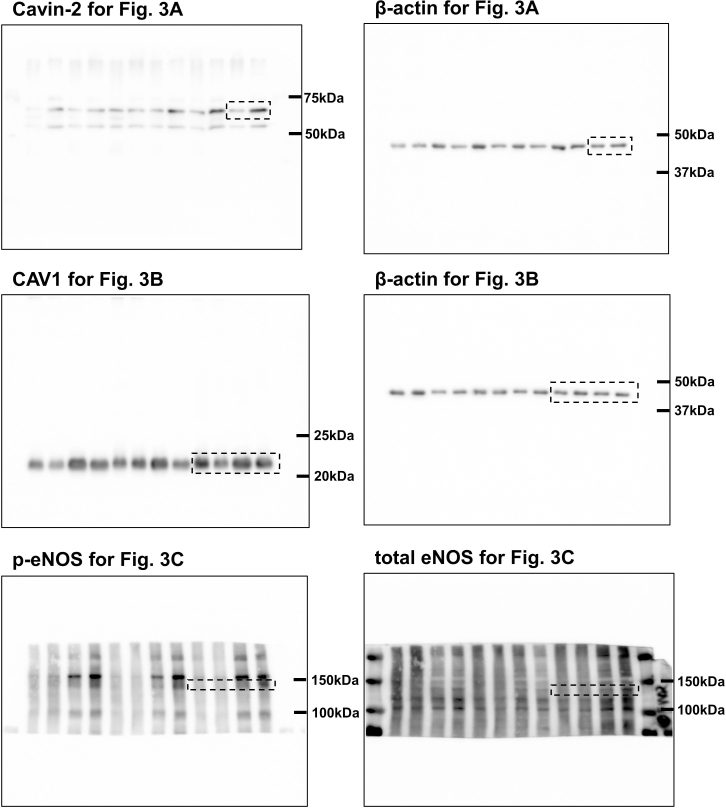
The original blots of Cavin-2 and β-actin (Fig.3A), CAV1 and β-actin (Fig. 3B), p-eNOS and total eNOS (Fig. 3C).

**Fig. S8:**
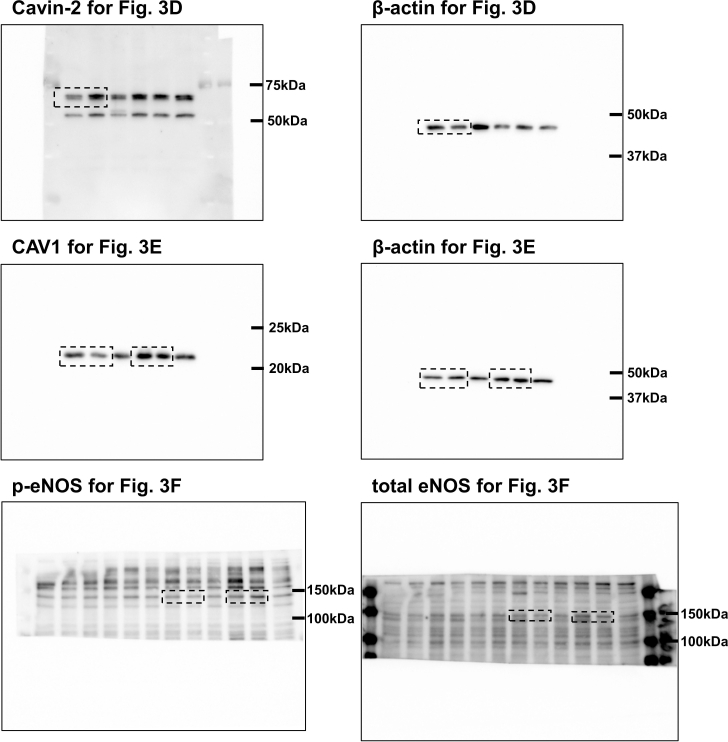
The original blots of Cavin-2 and β-actin (Fig.3D), CAV1 and β-actin (Fig. 3E), p-eNOS and total eNOS (Fig. 3F).

**Fig. S9:**
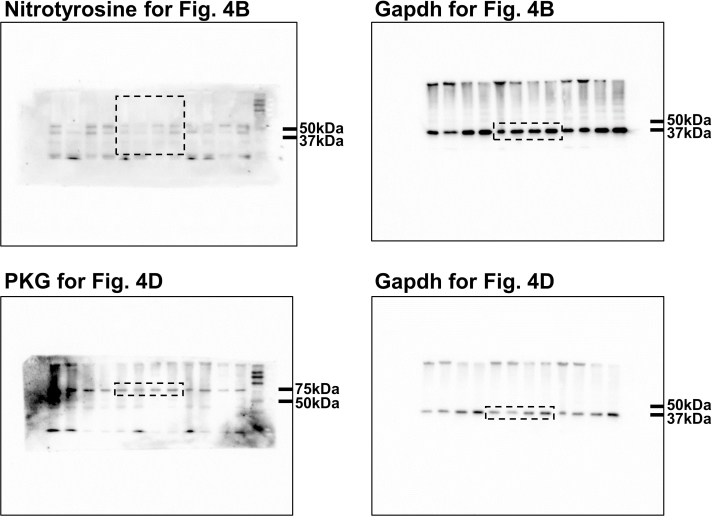
The original blots of Nitrotyrosine and Gapdh (Fig.4B), PKG and Gapdh (Fig. 4D).

PKG impairment accelerates the phosphorylation of myosin light chain (MLC) [25]. Smooth muscle cell tension is primarily determined by phosphorylation (contraction) and dephosphorylation (relaxation) of the regulatory MLC [26]. Therefore, we further evaluated the phosphorylation of MLC2 in the lung tissue by western blotting. MLC2 phosphorylation tended to be increased, but not significantly, in the Cavin-2 mice after hypoxia (Fig. S6).

## 4 Discussion

**Fig. 5:**
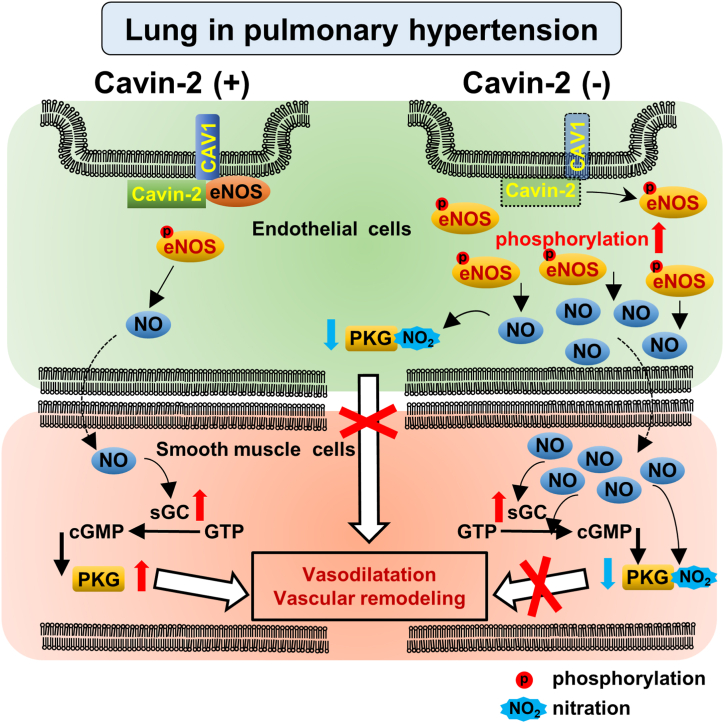
**Schematic presentation describing the possible mechanism by which Cavin-2 loss exacerbates hypoxia-induced pulmonary hypertension.** Cavin-2 stabilizes caveolin-1 and inhibits eNOS phosphorylation in endothelial cells. Loss of Cavin-2 decreases caveolin-1 in endothelial cells and increases phosphorylated eNOS in pulmonary hypertension. Excessive eNOS phosphorylation produces excessive NO and causes extensive nitration of intracellular proteins, including PKG in smooth muscle cells, impairing vasodilation and remodeling.

The present study showed that Cavin-2 loss exacerbates PH after hypoxic exposure. The Cavin-2 loss resulted in an excessive increase in phosphorylated eNOS in the lungs and HPAECs after hypoxic exposure, accompanied by a decrease in CAV1, suggesting that Cavin-2 cooperates with CAV1 in the regulation of eNOS phosphorylation in PH (Fig. 5).

An increase in phosphorylated eNOS is one of the mechanisms of PH development in CAV1 KO mice. CAV1 not only regulates caveolae formation in endothelial cells [8] but also suppresses eNOS activity in endothelial cells by binding to eNOS [9]. CAV1 loss causes excessive eNOS phosphorylation, and the resulting NOx overproduction promotes protein nitration, including in surrounding smooth muscle cells. This eNOS-induced nitration also affects PKG involved in vasodilation and vascular remodeling. Nitration of PKG decreases its activity in smooth muscle cells leading to worsening PH. In our study, Cavin-2 loss significantly decreased the amount of CAV1 in the mouse lungs and HPAECs and, at the same time, significantly enhanced NOx production and nitration. We also observed an increasing trend in the nitration of PKG in the lung. The present study could not directly demonstrate that Cavin-2 regulates eNOS hyperphosphorylation via the reduction of CAV1, which is a limitation of this study. However, our results suggest that Cavin-2 is strongly involved in stabilizing CAV1 in endothelial cells. The CAV1 stabilization is necessary to suppress excessive eNOS phosphorylation leading to PKG nitration in smooth muscle cells. A previous report showed that Cavin-2 knockdown destabilized eNOS in human umbilical vein endothelial cells [17]. However, the HPAECs we used in the present study surprisingly showed that Cavin-2 knockdown enhanced eNOS activity after hypoxic exposure. It is difficult to fully explain the discrepancy in the results of eNOS phosphorylation in each ECs due to differences in experimental conditions. Still, our results in vitro are consistent with those obtained with Cavin-2 KO mice.

In the present study, we were unable to fully demonstrate the involvement of PKG or MLC in PH associated with Cavin-2 loss. However, we were able to show that PKG, which is essential for the vasorelaxation mechanism, may be impaired by Cavin-2 loss-mediated nitration, leading to accelerate MLC phosphorylation. Previous papers reporting the relationship between PH and MLC phosphorylation evaluated PASMCs isolated from PH lungs [25,27,28], which differ from the experiments in our method. Additionally, the hypoxia-induced PH model is reversible, using it as a suitable model for the study of early PH [29,30]. We may need more severe lung injury models to assess the MLC phosphorylation in lung tissue. Moreover, PKG is expressed not only in SMCs but also in ECs [31]. Although the PKG in PASMCs is largely involved in the direct mechanism of vasorelaxation via MLC dephosphorylation, it is possible that the PKG of PAECs is also involved in vasorelaxation in cooperation with PASMCs [[31], [32], [33]]. In any case, nitration of PKG is very important in PH development. Therefore, further studies are needed to determine whether loss of Cavin-2 is involved in PKG nitration and MLC phosphorylation of PASMCs.

It seems strange that Cavin-2 increases *p*-eNOS under hypoxic conditions but does not affect the accumulation of *p*-eNOS under normoxic conditions. Unfortunately, it is difficult to explain this result in the present study fully. However, some members of the Cavin family are known to play crucial roles only under some loading conditions. For example, Cavin-4 mediates an increase in p-extracellular signal regulated kinase (ERK) after α-adrenoceptor stimulation but does not affect *p*-ERK in the unstimulated steady state [14]. Our findings are also supported by the fact that Cavin-2 KO mice give birth and develop normally under unstressed conditions.

The development of PH and the loss of caveolae are not necessarily linked. Loss of CAV1, strongly associated with PH, leads to loss of caveolae in PAECs [34]. However, the loss of Cavin-4 shows hypoxia-induced PH without altering the shape or number of caveolae in PASMCs [13]. In the present study, the loss of Cavin-2 reduced the number of caveolae under normoxic conditions, but hypoxia exposure induced PH without further reducing caveolae numbers. Taken together, our results and previous reports suggest that the development of PH does not necessarily require caveolae loss.

Our findings show that Cavin-2 loss is associated with PH but not a sufficient factor for PH development. CAV1 deficiency causes PH under normoxia [35], whereas PH associated with Cavin-2 loss requires hypoxic exposure. Although Cavin-2 loss caused a decrease in CAV1 even under normoxia in our study, eNOS phosphorylation and amounts of nitrated proteins in lung tissue were similar in WT and Cavin-2 KO mice. Since the level of eNOS phosphorylation in HPAECs under normoxic conditions was significantly lower than the level after hypoxic exposure, our results suggest that even the amount of CAV1 reduced by Cavin-2 loss is sufficient to suppress eNOS phosphorylation under normoxic conditions. The PH development associated with the loss of Cavin-2 function requires another factor, persistent hypoxic exposure.

The present study showed that Cavin-2 was increased under hypoxia. Elucidating the regulation of Cavin-2 in hypoxia may help understand the mechanism of PH development. The protein expression level of Cavin-2 was upregulated in the lung and HPAECs after hypoxic exposure Still, the mRNA expression level of Cavin-2 was not upregulated in the lung and HPAECs after hypoxic exposure. The mRNA expression level of Cavin-1, CAV1, and CAV2 in HPAECs was also unaffected by hypoxic exposure. On the other hand, it was revealed that the degradation of Cavin-2 protein was significantly suppressed under hypoxic conditons. Our results suggest that degradation of Cavin-2, which has a CAV1 anchoring function, is inhibited under hypoxia and that Cavin-2 retains CAV1 on caveolae as much as possible. Cavin-2-mediated CAV1 retention may prevent excessive eNOS phosphorylation and suppress progression to PH.

In conclusion, this study clarified that Cavin-2 loss exacerbated hypoxia-induced PH. Cavin-2 is essential for maintaining CAV1 in endothelial cells and suppressing excessive eNOS phosphorylation. Cavin-2 loss promotes excessive eNOS phosphorylation under hypoxia via reducing CAV1, resulting in excessive nitration of lung tissue proteins, including PKG in smooth muscle cells. Although HPAECs did not give completely comparable results to mouse lung tissue, our results provide meaningful information on the inhibitory effect of Cavin-2 deficiency on hypoxia-induced PH and the mechanisms that constitute the PH. Unlike CAV1, there have been no reports that Cavin-2 is involved in human PH, but functional defects in Cavin-2 are a potential risk of developing PH.

## Author contribution statement

Takeru Kasahara: Performed the experiments; Analyzed and interpreted the data; Wrote the paper.

Takehiro Ogata: Conceived and designed the experiments; Performed the experiments; Analyzed and interpreted the data; Contributed reagents, materials, analysis tools or data; Wrote the paper.

Naohiko Nakanishi: Performed the experiments; Analyzed and interpreted the data.

Shinya Tomita, Yusuke Higuchi, Naoki Maruyama, Tetsuro Hamaoka: Performed the experiments.

Satoaki Matoba: Conceived and designed the experiments; Contributed reagents, materials, analysis tools or data.

## Funding statement

Dr Takehiro Ogata was supported by 10.13039/501100001691Japan Society for the Promotion of Science, Grants-in-Aid for Scientific Research (JSPS KAKENHI) {JP18K07046}.

## Data availability statement

Data included in article/supp. material/referenced in article.

## Additional information

No additional information is available for this paper.

## Declaration of competing interest

The authors declare that they have no known competing financial interests or personal relationships that could have appeared to influence the work reported in this paper.
