# Supplementary material for: Cavin-2 loss exacerbates hypoxia-induced pulmonary hypertension with excessive eNOS phosphorylation and protein nitration
Source: Heliyon. 2023 Jun 11;9(6):e17193. doi: 10.1016/j.heliyon.2023.e17193 (PMC10285171; doi:10.1016/j.heliyon.2023.e17193)
Supplement: Table S1 [file mmc1.docx]

**Table S1. The list of primer sequences used for RT-qPCR**

| **Gene** | **Forward primer** | **Reverse primer** |
| --- | --- | --- |
| **Mouse** |  |  |
| *Cavin-1* | 5′-CAGCGTCAACGTGAAGACC-3′ | 5′-CCTCCGACTCTTTCAGCGAC-3′ |
| *Cavin-2* | 5′- ATGAGGAAGCCCTGGAAGAT -3′ | 5′- CCCAGATGATGCTTTCTGGT -3′ |
| *GAPDH* | 5′-TTGTGATGGGTGTGAACCACGAGA-3′ | 5′-CATGAGCCCTTCCACAATGCCAAA-3′ |
| **Human** |  |  |
| *CAV1* | 5′-GCGACCCTAAACACCTCAAC-3′ | 5′-ATGCCGTCAAAACTGTGTGTC-3′ |
| *CAV2* | 5′-AAGACCTGCCTAATGGTTCTGC-3′ | 5′-CTCGTACACAATGGAGCAATGAT-3′ |
| *Cavin-1* | 5′-GAGGACCCCACGCTCTATATT-3′ | 5′-CCCCGATGATTTTGTCCAGGA-3′ |
| *Cavin-2* | 5′-CATCCGGGACAACTCACAGG-3′ | 5′-CAGCGTCTAGCATGTTCACCA-3′ |
| *β-actin* | 5′-CATGTACGTTGCTATCCAGGC-3′ | 5′-CTCCTTAATGTCACGCACGAT-3′ |
